# Peritoneal Dialysis of Three-Cuff Catheter Experience in Qassim Province, Saudi Arabia

**DOI:** 10.1155/2024/5554703

**Published:** 2024-10-30

**Authors:** Ahmed AlSalloom, Chandra Sekhar Kalevaru, Kholoud Alomeri, Sultan Alsayegh

**Affiliations:** ^1^King Fahd Specialist Hospital Buraidah, Buraydah, Al Qassim, Saudi Arabia; ^2^Family Medicine Academy, Buraydah, Al Qassim, Saudi Arabia

**Keywords:** three-cuff PD catheter, complications, KFSHB, Qassim, reinsertion of catheter, Saudi Arabia, time of therapy

## Abstract

**Background:** The global rise in noncommunicable diseases, including chronic kidney diseases (CKDs), has led to a significant increase in the use of dialysis units to enhance patient longevity and quality of life. Over time, two-cuff catheters have been replaced by three-cuff catheters, with their usage expanding in nephrology centers across Saudi Arabia. This study aimed to evaluate the benefits, complications, and duration of therapy associated with three-cuff catheters in peritoneal dialysis (PD) patients.

**Methodology:** To ensure the reliability of our results, we conducted a comprehensive cross-sectional study involving 257 patients who underwent three-cuff PD catheter (PDC) insertion and omentopexy. Data were retrospectively collected from 2016 to 2023 at King Fahad Specialist Hospital, Buraidah. The questionnaire was designed based on available variables in the records section and validated by subject experts and experienced research faculty. Data were then entered and analyzed using SPSS version 21.0. Descriptive statistics were employed for inferential statistics, while the chi-square test and logistic regression analysis were used to identify predictors of PD outcomes.

**Results:** The average duration of therapy was 27.84 months, with a standard deviation of 27.23 months. Early complications were minimal, with just 5.1% (*n* = 13) experiencing peritonitis, 0.8% (*n* = 2) facing catheter migration, and 0.4% (*n* = 1 each) encountering omental wrap and exit site infection (ESI) within 30 days of catheter insertion. Remarkably, only 7.8% (*n* = 20) required catheter reinsertion. In addition, catheter removal due to catheter-related issues was low, affecting only 3.8% of patients.

**Conclusions:** According to the study findings, three-cuff catheters exhibited fewer complications, superior performance, and longer therapy duration. These outcomes may be attributed to the thorough design of the three-cuff catheters, the dedication of the staff, and the implementation of strict policies. To maintain these positive results, it is crucial for the Ministry of Health and the Health Cluster to adopt long-term supportive measures.

## 1. Introduction

In 2008, only two patients utilized peritoneal dialysis (PD) services in Qassim province. By 2023, the number of cases had greatly improved, touching 257 patients, according to King Fahad Specialist Hospital Buraidah statistics. Before 2016, two-cuff catheters were used in Qassim, and after that, three-cuff catheters were introduced in our institute for treating PD patients.

PD was first introduced in 1923, and by 1950, it had been used to treat and review 100 patients. Today, its use has significantly increased in nephrology units and is recognized as an effective therapy for patients with end-stage renal disease (ESRD) [1, 2]. The three-cuff PD catheter (PDC) is typically placed in the pelvis, ensuring the omentum and exit site are visible and positioned slightly away from the belt line. While the length, design, and insertion type of the catheter each have their benefits, no single insertion method is considered superior [3, 4]. However, catheter tip migration is a common complication that can impair catheter function [5]. A study in Al-Khobar reported that a new triple-cuff PDC resulted in less migration and fewer entry site infections [6].

### 1.1. Advantages of PD

Compared to hemodialysis, PD offers better control of hypertension, diet, fluid balance, and anemia, and it eliminates the need for dialysis machines [1]. This procedure can be performed at home, reducing costs, travel, and allowing patients to remain in a familiar environment with family.

### 1.2. Common Complications

Most of the studies observed a common complication in PD, and also in Riyadh [1], the study highlighted that peritonitis is the most common complication: tip migration, which significantly impairs catheter function. Migration of the catheter tip was observed in 10%–35% [7, 8]; this could be due to the PD technique of all PD patients, which leads to impaired fluid drainage. To overcome this issue, several techniques, modifications, and interventions were developed to prevent PDC migration [6].

A 2018 study in Riyadh on PD infections in tertiary care hospitals involved 100 patients. The mean duration of PD was 28.05 months, with hypertension being the most common cause of ESRD. Among these patients, 45 developed technique-related infections (TRI) and 12 developed non-TRI (NRTI). The study found a significant difference between patients with TRI and the presence of diabetes and duration of dialysis, with 18 patients switching to hemodialysis [9].

A 2013 study in Central Saudi Arabia, using retrospective data from 2006 to 2011 at King Saud Medical City, Riyadh, found that the mean age of patients was 51.2 ± 14.5 years. Diabetic nephropathy was the most common complication of ESRD, with 27.5% of dialysis patients experiencing complications. The most common issues were peritonitis (9.2%) and mechanical dysfunction (8.6%) [10].

An international study in Jordan involving 269 patients (both hemodialysis and PD) over 10 years (2009–2019) reported that six patients permanently switched to hemodialysis. Peritonitis was a common complication (52.5%), along with exit site infections (ESI) (30%) and catheter malfunction (12.5%) [11]. Due to the aforementioned conditions and factors, this study was designed to investigate the duration of therapy, complications such as catheter migration, and related comorbidities among PD patients at our institute.

## 2. Objectives

The objectives of PD are as follows:

1. To detect the benefits and complications of three-cuff catheters about mechanical and infectious nature
2. To estimate the relation to time on therapy (TOT) and catheter replacement frequency with three-cuff PDC
3. To find the demographic characteristics, reinsertion, and complications status and its association with TOT among PD patients in Qassim province

## 3. Patients and Methods

Study setting: King Fahad Specialist Hospital Buraidah (KFSHB) is a tertiary care center that provides specialist services for the people of Qassim province.

Study design: A record review study was conducted in the Medical Record section of King Fahad Specialist Hospital Buraidah.

Definitions adopted in the study (As per the International Society for PD) are as follows:

Medication adherence: Adherence criteria were taken from the patient as a subjective word.

Diet adherence: This is based on controlled phosphate and less salt consumption.

Anemia control: Patients with hemoglobin less than 10 g/dL are considered uncontrol, and those with a range from > 10 to 13 g/dL are considered control.

Blood pressure control: The patient's blood pressure of 130/80 mm of hg is considered a control status.

Phosphate control: < 1.79 mmol/L is considered as a control.

Body fluid control: Body weight equal to dry weight and no clinical signs of overload.

Adequacy control: kt/v is 1.7 as control (where k-clearance, t-time, and v – volume).

PTH control: three to nine times the normal parathyroid hormone (PTH) level is considered as control.

### 3.1. Questionnaire

The questionnaire was developed based on previous studies conducted in Saudi Arabia and globally [1, 6, 11]. Based on our objectives, the study was designed and a questionnaire was developed. After completing the meeting with the subject expert and research colleague, I visited the medical records section at KFSHB to identify the required variables' availability and another feasibility point of view. The questionnaire tool was developed once the questionnaire was prepared and validated with peer colleagues and research experts.

The first part of our questionnaire included demographic variables such as the patient's age, gender, education, and occupation. The second part of the questionnaire covered specific points related to PD, the status of the patients' complications, and the success rate with PD. Certain infectious complications and mechanical complications were also considered in our study. In addition, the shift to hemodialysis and its reasons were also taken as variables to get a comprehensive picture of the PD experience in our province.

## 4. The Technique of the Triple Cuff Insertion With the Low-Entry Approach

A 57-cm triple-cuff PDC was used for all participants, according to the preparation guidelines, under anesthesia, following an aseptic procedure. Pneumoperitoneum was created using a Veress needle at a pressure of 10–15 mm Hg. A 5-mm port was inserted in the right hypochondriac region at the midclavicular line, 2 cm below the costal margin, for the 30-degree laparoscopic camera. A diagnostic laparoscopy was then performed to check for adhesions or herniations and to evaluate the omentum size. Omentopexy was performed at the surgeon's discretion if the omentum was long and redundant [12].

**Figure 1:**
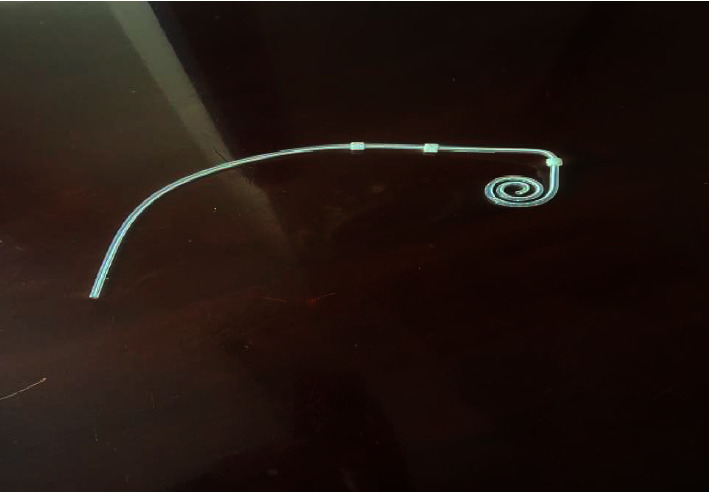
Three-cuff Saudi catheter.

The three-cuff PDC was inserted, the catheter tip was positioned in Douglas's pouch, and the catheter was advanced so that the distal cuff was located at the anterior surface of the rectus muscle. A subcutaneous tunnel was created obliquely lateral to the umbilicus. The second cuff was positioned about 10 cm from the distal cuff, and the proximal cuff was 2 cm from the exit site [12]. At the end of the operation, 500 mL of PD fluid was installed through the PDC to confirm catheter patency and the free inflow and outflow of the PD solution. At the end of the procedure, 500 mL of PD fluid was instilled through the PDC to confirm its patency and ensure free inflow and outflow of the dialysis solution. The final position of the three-cuff PDC is shown in Figure 1.

Sample size: All the PD patients since inception at KFSH were considered as a sample.

Sampling method: No sampling method was applied as we took all the PD patients in our department.

Study duration: From the research proposal to the research completion, 1 year is tentative.

### 4.1. Inclusion Criteria

All the PD patients with 1-year experience were included.

### 4.2. Exclusion Criteria

Those patients taking alternative dialysis other than PD were excluded.

Primary peritonitis was defined as peritonitis within 1 month of PDC insertion, while secondary peritonitis was beyond 1 month [13].

### 4.3. Ethical Considerations

After the research proposal was prepared, the Institutional Ethical Committee's approval was received, with approval number 607-45-4119. Concerned medical records were in charge of obtaining permission, which was taken from the medical director at KFSHB, Buraidah. Privacy and confidentiality of individual information were maintained throughout the research.

### 4.4. Pilot Study

After the Institutional Review Board's approval, a pilot study was conducted on 10 records. After the pilot study's completion, no changes were initiated in the study design and feasibility.

### 4.5. Statistical Analysis

Data were entered, coded, and analyzed using the SPSS 21.0 version. Descriptive statistics were calculated for the demographic factors. For the categorical analysis, the chi-square test was applied. Linear regression analysis was applied with TOT-dependent variable with demographic factors (age, gender, education, marital status, and occupation), reinsertion of the PDC, and complications status. A probability (P) value less than or equal to 0.05 for all the statistical inferences was considered for the significance.

## 5. Results

In the present study, 257 patients were retrieved from the patient files of the Nephrology Department from the period 2016 to 2023. The study population's mean age and standard deviation was 42.0 ± 15 years (Table 1). About 28% were from the < 30 years of age group. Females comprised 46.3% (*n* − 119), and close to one-fourth of the people (24.5%, *n* − 63) were from government occupations. The mean TOT and SD in the study population was 27.84 ± 27.23 months. The range of OPD visits was from 10 to 890. In the study population, about 63.4% were married, 30.4% were single, 5.1% were widowed, and 1.2% were divorced. The study group's education was 100% literacy. About 65% studied primary and secondary. 31.2% were college-level, and only 0.8% completed postgraduate studies. In the current study, about 92.6% (*n* − 238) of PD patients had comorbid conditions, and the remaining 7.4% (*n* − 19) had no comorbidity. The most common comorbid condition among PD patients was hypertension (59.9%, *n* − 154), followed by hypertension and diabetes (22.2%, *n* − 57).

Table 2 depicts that out of 257 PD patients, the most common complication observed among PD patients was 5.1% peritonitis (*n* − 13) within 1 month of catheter insertion. We also observed peritonitis rate 1 month after catheter insertion in PD patients (9.7%, *n* − 25). Only 4.7% (*n* − 7) had other complications following catheter insertion after 1 month. About 20 patients have undergone catheter insertion. Of which only three persons used two catheters. PDC migration was rare, accounting for only 0.8% (*n* − 2), but these two patients had some surgical difficulties during insertion.

Table 3 shows that only 3.8% of patients removed catheters due to catheter-related problems and 1.9% due to omental wrap.

Table 4 denotes that among the PD patients, about 86.8% (*n* − 223) of medication adherence and 64.6% (*n* − 166) of them adhered to diet. Anemia control was 220 (85.6%) and blood pressure control was 213 (81.9%). Patients who underwent parathyroidectomy were 4.7%.

Table 5 stated that statistically significant associations were observed with higher education and other occupations, which had a higher duration of therapy with PD (*P* *value* 0.038 and *P* *value* 0.014), respectively. Patients who had reinsertion had 38.5 times more duration of TOT than patients who did not undergo catheter reinsertion (*P* *value* 0.001). The mean duration of therapy among reinserted patients (*n* − 20, 7.7%) was 52.60 ± 52 months. However, patients with complications had 7.9 times less duration of TOT with PD patients (*P* *value* 0.045).

## 6. Discussion

In Qassim province, King Fahad Specialist Hospital offers tertiary care and provides free dialysis services to all Saudi and governmental populations. This initiative promotes awareness through predialysis education for chronic kidney disease (CKD), as noted in a study from Jeddah [14]. A cross-sectional record review study was conducted from 2023 to 2024, involving 257 patients admitted to the Nephrology unit at KFSH. The study population had a mean age of 42.0 ± 15 years, similar to a study in Riyadh, which reported a mean age of 40.7 ± 19.3 years [9].

In the current study, the mean TOT was 27.84 ± 27.23 months. An observational study at the University Hospital of Saudi Arabia, involving 153 patients over three years, reported a mean TOT of 15.4 ± 5.8 months with automated PD (APD) [15]. The shorter TOT in their study could be due to the limited duration of experience. Another study in India found that the mean survival of two-cuff catheters ranged from 18.63 ± 15.07 months to 16.73 ± 7.84 months among surgeons and nephrologists [13]. The higher TOT in our study may be attributed to factors such as the use of three-cuff PDC, monthly follow-ups, and a rigorous training program.

Among the 257 PD patients in our study, the most common complication within 1 month of catheter insertion was peritonitis, affecting 5.1% (*n* = 13) of patients. Beyond 1 month, peritonitis was observed in 9.7% (*n* = 25) of patients. The International Society of PD (ISPD) recommends annual audits of catheter-related complications to improve practice and states that peritonitis rates within 30 days of insertion should be less than 5% [5, 16]. Our study's peritonitis rate is low, possibly due to the use of a three-cuff PDC and our sophisticated unit policy for patient training and retraining.

A study in India comparing PDC insertion by surgeons and nephrologists found no significant difference in peritonitis rates between the two groups. However, both groups experienced common complications related to the PDC insertion technique. Primary peritonitis occurred in two patients in the surgeon group and none in the nephrologist group, while secondary peritonitis was observed in 9.6% of nephrologists' patients and 11.6% of surgeons' patients [13].

In our study, 4.7% (*n* = 7) of patients experienced other complications after 1 month of catheter insertion. ESI was observed in 2.7% (*n* = 7) of patients over 7 years. A survey in the United States of America recommended that the exit site/tunnel infection rate should be less than 5% within 30 days of catheter insertion [5, 16]. Prolonged ESI can lead to tunnel infection and peritonitis, indirectly increasing PD failure rates [17, 18]. A study in Norway reported that despite adequate antibiotic coverage, two out of 38 active PD patients were reinfected [19].

In the current study, catheters were removed in only 3.8% of patients due to catheter-related issues. A 15-year retrospective analysis in Taiwan found that 10.5% of catheters were removed due to obstruction, with 1.9% due to omental wrap [17]. A study in China identified omental wrapping as a common complication in PD users, often leading to catheter migration and outflow failure [20]. Our study observed fewer catheter removals, likely due to the use of three-cuff catheters and omentopexy procedures.

In this study, 92.6% of patients had comorbid conditions, with hypertension being the most common, affecting 59.9% of PD patients. A systematic review in Korea found that 70%–80% of PD patients had hypertension [21], while an Italian multicentric study reported a prevalence of 88.1% [22].

Anemia control was achieved in 85.6% of patients in our study. Various studies have shown that anemia is commonly associated with dialysis patients and significantly impacts their quality of life, including mortality and morbidity in CKD patients [23, 24]. Research in Brazil indicated that 28% of PD patients had anemia, defined as a hemoglobin level of 11 g/dL [25].

Our study found that 86.8% (*n* = 223) of patients adhered to their medication regimen. A systematic review in Singapore reported that nonadherence among PD patients ranged from 3.9% to 85% [26]. Similarly, an Australian study noted that nonadherence to therapy was common among dialysis patients, potentially leading to higher mortality and adverse outcomes in ESRD patients [27].

Blood pressure control was observed in 81.9% (*n* = 213) of patients. Effective blood pressure management is crucial for reducing cardiovascular-related mortality in PD patients. A systematic review at the University Hospital highlighted that good hypertension control lowers mortality in PD patients [28]. Some limitations of our study include its retrospective nature and lack of comparative results within our center. However, its strengths lie in the extensive seven-year data collection (2016–2023) and the large sample size.

## 7. Conclusions

In conclusion, the study findings indicate that three-cuff catheters resulted in fewer complications and a lower rate of catheter removal, leading to longer therapy durations. Our study also demonstrated effective control of anemia, hypertension, fluid balance, and medication adherence among PD patients. These results suggest that administrative staff and policymakers should advocate for the use of three-cuff catheters and enhance training and retraining programs.

## Figures and Tables

**Table 1:** Demographic characteristics of the PD patients at KFSH, Qassim.

Nationality	Number of participants	Percentage
Age ± SD	42.0 ± 15 years	
Age category		
14–30 years	72	28.0
31–50 years	104	40.5
> 50 years	81	31.5
Gender		
Male	138	53.7
Female	119	46.3

**Table 2:** Complication status, catheter insertion, and postsurgical complications among PD patients.

Variables	Yes (%)	No (%)
Complications status	77 (30.0)	180 (70.0)
Reinsertion of the catheter	20 (7.8)	237 (92.2)

**Catheters insertion**	**Number**	**Percentage**

Number of catheters: 1	17	6.6
2 catheters	3	1.2
Not inserted catheters	237	92.2

**Postsurgical complication (within 30 days of catheter insertion)**	**Number**	**Percentage**

Omental wrap	1	0.4
Exit site infection (ESI)	1	0.4
Others	4	1.6
Peritonitis	13	5.1
Subcutaneous leakage	1	0.4
Not applicable (NA)	237	92.2

**Late complications (after 1 month of catheter insertion):**	**Number**	**Percentage**

ESI	7	2.7
Migration	2	0.8
Omental wrap	5	1.9
Pericardial effusion	1	0.4
Peritonitis	25	9.7
Pleural leakage	3	1.2
Subcutaneous leakage	6	2.3
Others	12	4.7
Not applicable (NA)	194	75.5

**Table 3:** Catheter removal due to catheter-related causes in the study population.

Variables	Yes (%)	No (%)
Catheter removal	10 (3.8)	247 (96.2)

**Reason for catheter removal (*n* − 10)**	**Number**	**Percentage**

Catheter not working	4	1.6
Intestinal adhesion	1	0.4
Omental wrap	5	1.9

**Table 4:** Patient medication, clinical and biochemical parameters status among PD patients.

Variables	Yes (%)	No (%)
Medication adherence	223 (86.8)	34 (13.2)
Diet adherence	166 (64.6)	91 (35.4)
Anemia control	220 (85.6)	37 (14.4)
Blood pressure control	213 (81.9)	44 (18.1)
PTH control	124 (48.2)	133 (51.8)
Parathyroidectomy	12 (4.7)	145 (95.3)
Serum phosphate control	167 (65)	90 (35)
Body fluid control	194 (75.5)	63 (24.5)
Adequacy control	184 (71.6)	73 (28.4)

**Table 5:** Linear regression analysis of demographic and other variables with time on therapy of peritoneal dialysis patients.

Continuous variable	Cat. Variables	Beta (B)	*p* value	Confidence interval (CI)
Time on therapy	Age 14–30 years	0.439	0.857	−4.355 to 5.233
	31–50 years			
	> 50 years			

Time on therapy	Gender: male	6.424	0.106	−1.385 to 13.232
	Female			

Time on therapy	Married	−0.997	0.709	−6.265 to 4.271
	Single			
	Widowed			
	Divorced			

Time on therapy	Primary education	5.632	0.038	0.308 to 10.957
	School education			
	College level			
	Postgraduation			

Time on therapy	Occupation: Govt.	3.915	0.014	0.797 to 7.034
	Private			
	Retired			
	Unemployed			

Time on therapy	No re-insertion	38.571	0.001∗	26.056 to 51.086
	Reinsertion			

Time on therapy	No complication	−7.976	0.045∗	−15.788 to −0.164
	Complication			

*Note:* Reference category: first category of each categorical variable.

^∗^Statistically significant.

## Data Availability

The data are not publicly available due to privacy or ethical restrictions. All data analyzed during the study are included within the article. Additional data can be shared upon request from the editor, reviewers, or any concerned authority. For further inquiries, please contact the corresponding author.
